# Neuroprotective effect of therapeutic hypothermia versus standard care alone after convulsive status epilepticus: protocol of the multicentre randomised controlled trial HYBERNATUS

**DOI:** 10.1186/s13613-016-0159-z

**Published:** 2016-06-21

**Authors:** Stephane Legriel, Fernando Pico, Yves-Roger Tran-Dinh, Virginie Lemiale, Jean-Pierre Bedos, Matthieu Resche-Rigon, Alain Cariou

**Affiliations:** Medical-Surgical Intensive Care Unit, Centre Hospitalier de Versailles - Site André Mignot, 177 rue de Versailles, 78150 Le Chesnay Cedex, France; INSERM U970 (Team 4), Paris Cardiovascular Research Center, Paris, France; Neurology and Stroke Department, Centre Hospitalier de Versailles - Site André Mignot, 177 rue de Versailles, 78150 Le Chesnay Cedex, France; Neurophysiology Department, Hôpital Lariboisière, AP-HP, 2 rue Ambroise Paré, 75010 Paris, France; Medical Intensive Care Unit, Saint Louis University Hospital, AP-HP, Paris, France; SBIM Biostatistics and Medical information, Hôpital Saint-Louis, APHP, 1 avenue Claude Vellefaux, Paris, France; Université Paris Diderot, Paris, France; ECSTRA Team (Epidémiologie Clinique et Statistiques pour la Recherche en Santé), UMR 1153 INSERM, Université Paris Diderot, Sorbonne Paris Cité, Paris, France; Medical Intensive Care Unit, Cochin University Hospital, Hopitaux Universitaires-Paris Centre, AP-HP, Paris, France; Paris Descartes University, Sorbonne Paris Cité–Medical School, Paris, France

**Keywords:** Therapeutic hypothermia, Randomised controlled trial, Status epilepticus, Outcome, Intensive care unit

## Abstract

Convulsive status epilepticus (CSE) is a major medical emergency associated with a 50 % morbidity rate. CSE guidelines have recommended prompt management for many years, but there is no evidence to date that they have significantly improved practices or outcomes. Developing neuroprotective strategies for use after CSE holds promise for diminishing morbidity and mortality rates. Hypothermia has been shown to afford neuroprotection in various health conditions. We therefore designed a trial to determine whether 90-day outcomes in mechanically ventilated patients with CSE requiring management in the intensive care unit (ICU) are improved by early therapeutic hypothermia (32–34 °C) for 24 h with propofol sedation. We are conducting a multicentre, open-label, parallel-group, randomised, controlled trial (HYBERNATUS) of potential neuroprotective effects of therapeutic hypothermia and routine propofol sedation started within 8 h after CSE onset in ICU patients requiring mechanical ventilation. Included patients are allocated to receive therapeutic hypothermia (32–34 °C) plus standard care or standard care alone. We plan to enrol 270 patients in 11 ICUs. An interim analysis is scheduled after the inclusion of 135 patients. The main study objective is to evaluate the effectiveness of therapeutic hypothermia (32–34 °C) for 24 h in diminishing 90-day morbidity and mortality (defined as a Glasgow Outcome Scale score <5). The HYBERNATUS trial is expected to a decreased proportion of patients with a Glasgow Outcome Scale score lower than 5 after CSE requiring ICU admission and mechanical ventilation.

*Trial registration* Clinicaltrials.gov identifier NCT01359332 (registered on 23 May 2011)

## Background

Convulsive status epilepticus (CSE) is a life-threatening emergency that requires on-scene antiepileptic treatment adjusted according to the treatment response [1]. Hospital mortality is about 20 % overall and reaches 40 % in patients with severe refractory CSE [2]. The morbidity associated with CSE is more difficult to evaluate, as adverse events are often erroneously attributed to the cause of CSE. In retrospective studies, Glasgow Outcome Scale (GOS) scores indicated neurological impairments in 28 % of patients after CSE and 54 % after refractory CSE [3]. In a prospective multicentre study, 50 % of CSE survivors requiring ICU admission had impaired 90-day functional outcomes defined as a GOS score <5 [4]. By multivariate analysis, factors independently associated with poorer outcomes were age, duration of motor seizures, focal neurological signs, and progression to refractory status epilepticus (SE) [4]. Some of these factors may be amenable to improvement and therefore may constitute therapeutic targets. Thus, outcomes may be improved by the rapid control of seizure activity (shorter seizure duration and prevention of refractory CSE and NCSE) and by treatment of the cause of CSE [5, 6].

Therapeutic hypothermia has shown both anticonvulsant and neuroprotective properties after SE in experimental studies. In rats, hypothermia at 32–34 °C decreased the incidence of both seizures and associated local and hippocampal neuronal damage [7–13]. In humans, therapeutic hypothermia has been described merely as an adjuvant treatment of super-refractory SE in adults and paediatric patients [14–17]. Finally, therapeutic hypothermia may also target some of the causes of status epilepticus such as ischaemic or haemorrhagic stroke [18–22] and subarachnoid haemorrhage [23–25]. Its use in patients with traumatic brain injury demonstrated conflicting results [26–30]. Thus, pathophysiological considerations and experimental data suggest a beneficial effect of therapeutic hypothermia in SE. Proof of such an effect in human patients has not been obtained [31, 32].

The main goal of the HYBERNATUS trial is to determine whether therapeutic hypothermia (32–34 °C) for 24 h has neuroprotective effects compared to standard care alone. HYBERNATUS is a multicentre, open-label, parallel-group, randomised, controlled trial in patients with CSE requiring admission to the intensive care unit (ICU) and mechanical ventilation.

## Methods/design

### Ethical aspects

The study protocol was approved by the local independent ethics committee (Comité de Protection des Personnes CPP Ile de France IV, Saint Louis, on 1 September 2010, #2010/27) and the competent French health authorities (AFSSAPS, on 22 June 2010, #EudraCT 2010-A00466-33). Informed consent is obtained from each patient or next of kin prior to study inclusion. The trial was registered at Clinicaltrials.gov identifier NCT01359332 (registered on 23 May 2011).

### Aims

The main study objective is to evaluate the effectiveness of therapeutic hypothermia (32–34 °C) for 24 h in decreasing 90-day morbidity and mortality, defined as a GOS score <5, in patients with CSE requiring ICU admission and mechanical ventilation.

The secondary objectives are to evaluate the effectiveness of therapeutic hypothermia (32–34 °C) for 24 h in decreasing seizure duration, progression to refractory CSE, ICU mortality, hospital mortality, 90-day mortality, hospital stay length, and 90-day functional impairments (motor and/or cognitive impairment, epilepsy).

### Design

HYBERNATUS is a multicentre, open-label, parallel-group, randomised, controlled trial in patients with CSE requiring ICU admission and mechanical ventilation. Patients are allocated at random to either early therapeutic hypothermia plus standard care or standard care alone at the acute phase of ICU management.

### Setting

Among the 11 French ICUs participating in the study, 6 are in university hospitals. All participating ICU staff members have received training in the study procedures and in protocols for optimal CSE management.

### Study population

Inclusion criteria are age over 18 years, admission to one of 11 participating ICUs, CSE (5 min or more of continuous clinical seizure activity or more than two seizures without a return to baseline in the interval), time since seizure onset no longer than 8 h, and need for mechanical ventilation.

Exclusion criteria are a full recovery, need for emergent surgery (neurosurgery or other) precluding therapeutic hypothermia, postanoxic status epilepticus, dying patient, decision to withdraw or withhold life-sustaining treatments, and pregnancy.

All patients admitted to one of the participating ICUs with any seizure activity are screened for eligibility by the ICU physicians 24/7.

### Randomisation

Consecutive eligible patients are randomly allocated in a 1:1 ratio to one of the two treatment groups. The randomisation scheme involves permuted blocks and stratification on centre, age (≤65 or >65 years), and seizure duration (≤60 min or >60 min). Randomisation and concealment are ensured by using a secure, computer-generated, interactive, response system available at each study centre and managed by the biometrics unit of the Saint Louis University Hospital (Paris, France), which has no role in patient recruitment.

### Study interventions

**Fig. 1:**
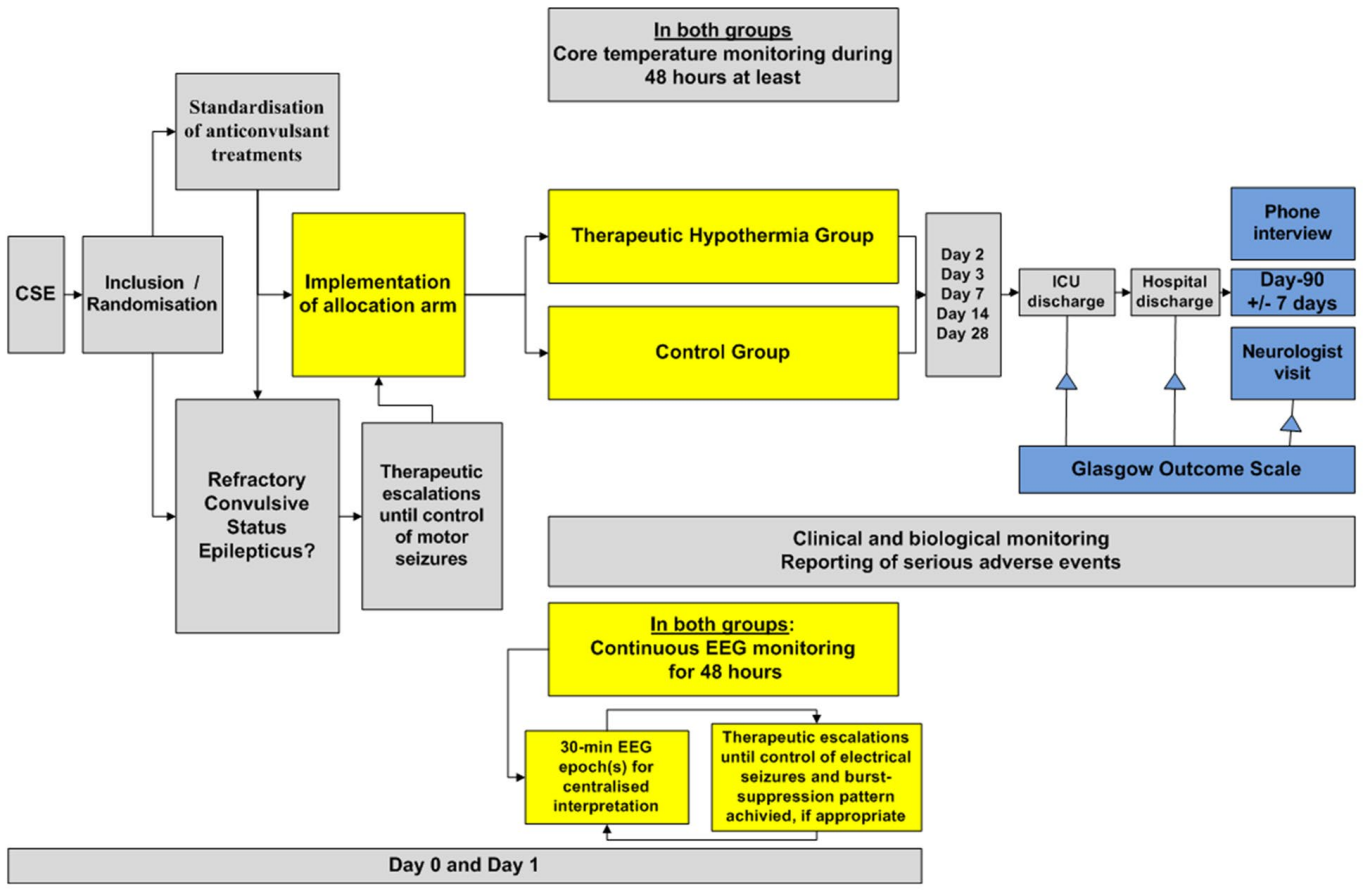
HYBERNATUS trial diagram. *ICU* intensive care unit. *EEG* electroencephalogram

**Fig. 2:**
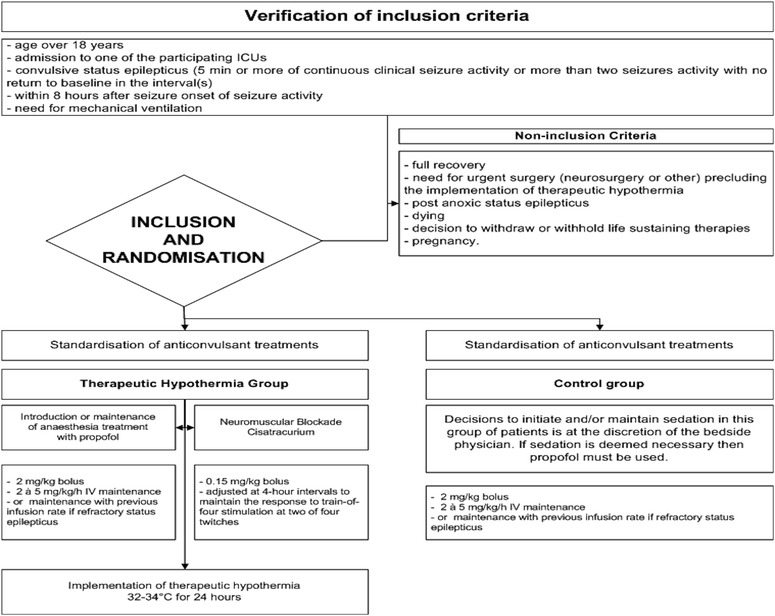
Eligibility criteria, inclusion, randomisation, and treatment implementation modalities in each arm of the trial

The study protocol and randomisation arms are detailed in Figs. 1 and 2 and Table 1.

**Table 1:** HYBERNATUS trial flow chart

	Inclusion	Day 0	Day 1	Day 2 Day 7 Day 14 Day 28	ICU discharge	Hospital discharge	Day 90 (±7 days)
Eligibility: check inclusion and non-inclusion criteria	X						
Patient/next of kin information and consent	X						
Randomisation (within 8 h after CSE onset)		X					
Standardised anticonvulsant treatment		X					
Brain imaging and other aetiological investigations		X					
Therapeutic hypothermia (in the intervention group)		X	X				
Core temperature monitoring (every 4 h)		X	X				
Continuous EEG monitoring		X	X				
30-min continuous EEG extract (centralised reading)		X	X				
Clinical and laboratory monitoring		X	X	X	X		
Serious adverse event reporting		X	X	X	X	X	X
Glasgow Outcome Scale (GOS) score evaluation					X	X	X^1, 2^
Standard EEG							X^2^
Brain MRI							X^2^
MMSE score							X^2^
FIM scale score							X^2^
Neurological evaluation							X^2^

^1^By phone interview in all cases

^2^Neurologist visit, if feasible

### Standardised anticonvulsant treatments after randomisation in both groups

To be eligible, patients should have received at least one of the first-line anticonvulsant drugs (clonazepam and diazepam) listed in French guidelines. Patients with ongoing motor seizures who have not received a second-line anticonvulsant (fosphenytoin, phenytoin, phenobarbital, or sodium valproate) before randomisation receive a loading dose of one of these drugs selected according to the indications and specific contraindications of each [33].

Patients with refractory CSE, defined as CSE persisting despite two lines of anticonvulsant therapy within 60 min, are given an intravenous 2 mg/kg propofol bolus followed by an additional intravenous 1 mg/kg bolus every 5 min until motor seizures stop, at which point a continuous intravenous propofol infusion is started at a rate of 2–5 mg/kg/h. In patients with uncontrolled refractory CSE despite continuous intravenous propofol at a rate of 5 mg/kg/h, anaesthetic drugs (midazolam or thiopental) can be added.

CSE must be controlled within 60 min after randomisation in both groups and before the induction of therapeutic hypothermia in the experimental group.

### Therapeutic hypothermia group

The objective is to lower the core body temperature to 32–34 °C rapidly after randomisation and then to maintain this temperature for 24 h. The management in the therapeutic hypothermia group will include the following:

- sedation with propofol in a 1–2 mg/kg bolus followed by a maintenance infusion at a rate of 2–5 mg/kg/h; patients with refractory CSE before randomisation receive the propofol infusion rate that was required to stop the motor seizures;
- neuromuscular blockade with a 0.15 mg/kg bolus of cisatracurium or of another neuromuscular blocker followed by doses at 4-h intervals adjusted to maintain the response to train-of-four stimulation at two of four twitches;
- hypothermia induction with ice-cold intravenous fluids at 4 °C and then hypothermia maintenance with ice packs at the groin and neck and a cold-air tunnel around the patient’s body;
- after 24 h of hypothermia, passive rewarming by covering the patient with a warm blanket, at a slow rate of 0.25–0.5 °C per hour to avoid haemodynamic instability, cisatracurium is discontinued when normothermia is achieved; when the train-of-four response is four twitches, the propofol is stopped or decreased to the lowest dose needed to ensure tolerance of the endotracheal tube;
- If continued sedation is required, the choice of the sedative is at the discretion of the bedside physician, but propofol is not used for longer than 48 h;
- Core body temperature is monitored using an oesophageal probe throughout the therapeutic hypothermia phase and then during rewarming until two values 4 h apart are between 37° and 38° C.

### Control group

The bedside physician decides whether sedation should be initiated and maintained. If sedation is given, the same propofol regimen as in the therapeutic hypothermia group is used (1–2 mg/kg bolus and then 2–5 mg/kg/h intravenous infusion rate). If sedation is inadequate with continuous intravenous propofol 5 mg/kg/h, benzodiazepines may be added. Finally, if sedation must be maintained beyond 48 h, the propofol must be stopped and an anaesthetic selected by the bedside physician given instead.

### General management of convulsive status epilepticus (CSE) in both groups

In both control and intervention groups, a continuous EEG monitoring is started within 2 h after randomisation and maintained during the first 48 h after randomisation and/or until body temperature normalisation in the therapeutic hypothermia group. A 30-min standard EEG extracted from the continuous EEG recording is sent for centralised interpretation by a qualified neurophysiologist within 2 h after the initiation of continuous EEG monitoring. If the results show refractory CSE or progression to NCSE, the patient is given a 2-mg/kg propofol bolus, repeated every 5 min if necessary, and then a maintenance intravenous infusion at 2–5 mg/kg/h to obtain and maintain a burst-suppression EEG pattern for 24 h. Further EEG epochs are sent for centralised interpretation by a qualified neurophysiologist until a burst-suppression EEG pattern is observed.

Second-line anticonvulsant drugs administered intravenously are switched to enteral administration when feasible. Patients with a known history of epilepsy are given their previous treatment enterally or parenterally. Aetiological investigations are carried out early, simultaneously with the symptomatic and anticonvulsant treatment. They include a thorough initial physical examination and tests for metabolic disturbances (e.g. hypoglycaemia, hypocalcaemia, hyponatraemia, uraemia, hypomagnesaemia, hypoxaemia, and hypercapnia) and carbon monoxide poisoning. Computed tomography (CT) of the brain is performed routinely. In patients with previously treated epilepsy, serum anticonvulsant drug assays are performed if appropriate, to determine whether levels are within the therapeutic range. As dictated by the clinical setting, tests are performed for disorders such as porphyria and thyroid dysfunction and/or for toxic substances (alcohol, cocaine, amphetamines, tricyclic, or serotonergic antidepressants). A lumbar puncture is performed in patients with any of the following: fever, meningeal neck stiffness, immunodeficiency, and negative findings from the other aetiological investigations. Magnetic resonance imaging (MRI) may be considered if all other aetiological investigations are negative [34].

### Other general measures

In both groups, a standardised neurological examination is performed every 4 h by the physicians and nurses in charge of the patient (GOS score, pupillary reflex, corneal reflex, cough reflex, spontaneous breathing, abnormal movements, focal neurological signs). Core body temperature is monitored continuously using an oesophageal probe and recorded every 4 h during the first 48 h after randomisation.

Patients are routinely screened for propofol infusion syndrome via measurements of arterial blood gas, serum lactate, serum triglycerides, and serum creatine phosphokinase every 8 h during the first 48 h after randomisation.

The prevention of secondary brain injury relies on temperature control to avoid hyperthermia in the control group and, after rewarming, in the therapeutic hypothermia group; glucose control between 0.8 and 1.4 g/L, maintenance of normoxia with PaO_2_ ≥ 80 mmHg and SaO_2_ ≥ 95 %; maintenance of normocapnia with PCO_2_ between 35 and 40 mmHg; 45° head-of-bed elevation; maintenance of mean arterial pressure between 70 and 90 mmHg; and serum sodium control between 138 and 142 mmol/L. Special attention is paid to the occurrence of aspiration pneumonia, and three samples for blood cultures are taken routinely within 24 h after randomisation.

### Safety and potential risks associated with the study

Rigorous monitoring procedures are implemented to minimise the risks associated with therapeutic hypothermia in patients after CSE. Continuous EEG monitoring is performed for 48 h after inclusion, and a 30-min EEG extract is interpreted centrally before implementation of the randomly allocated treatments. Although neuromuscular blockade can theoretically mask recurrent seizure activity, this risk is lessened by the anticonvulsant properties of therapeutic hypothermia.

Preliminary data show that 82 % of patients admitted to the ICU for CSE receive mechanical ventilation for longer than 24 h, and that nearly 25 % of those given mechanical ventilation for less than 24 h have a 90-day GOS score lower than 5. Thus, the use of mechanical ventilation for an unnecessarily long period seems very rare. No criteria are available for predicting which patients will require mechanical ventilation for less than 24 h. Finally, this study includes only patients with severe CSE requiring both ICU admission and mechanical ventilation.

Patients receiving therapeutic hypothermia and/or propofol are closely monitored for adverse effects of these treatments such as infections and propofol infusion syndrome. We therefore believe that the use of therapeutic hypothermia does not expose the patients to undue risks, given the high morbidity and mortality rate in our prospective study [4] and the safety measures included in the HYBERNATUS trial protocol.

### Data collection and follow-up

The following baseline characteristics are recorded at admission: age, sex, date and time of ICU admission, McCabe score and Knaus score, and pre-existing comorbidities. The Simplified Acute Physiology Score II is computed 24 h after ICU admission.

The study protocol also requires collection of the following data:

- Description of the CSE: circumstances of onset, date and time of onset and of seizure control, clinical features of seizures, pre-hospital and hospital providers and timing of anticonvulsant and supportive treatments, results of aetiological investigations (brain CT, lumbar puncture, MRI, laboratory tests including toxicological screen), aetiological diagnosis, type and dosage of antiepileptic drugs, and date and start and stop times of continuous EEG monitoring;
- Data related to ICU and hospital management: description of the acute disease; description of organ failures according to the Logistic Organ Dysfunction score (at inclusion and on days 0, 1, 2, 7, 14, and 28); use of mechanical ventilation, inotropic support, and/or renal replacement therapy; vital and functional status at ICU and hospital discharge according to the GOS; and cause of death (treatment limitation decision, refractory SE, and/or ICU complications);
- Data related to therapeutic hypothermia: modalities of induction and maintenance; core temperature every 4 h during the first 48 h; serum levels of creatine phosphokinase, triglycerides, and electrolytes; arterial lactate and blood gas levels; electrocardiogram; occurrence of aspiration pneumonia; and results of blood cultures on samples taken within the first 24 h;
- Data related to outcomes: 90-day GOS score by structured phone interview and neurologist visit; description of functional sequelae; mini–mental state examination (MMSE) score; and Functional Independence Measure scale (FIM) score.

### Organisation of the trial

#### Funding/support

HYBERNATUS is fully sponsored by the *Direction de la Recherche Clinique et du Développement* (DRCD), Assistance Publique—Hopitaux de Paris, France, with a grant from the French Ministry of Health (*Programme Hospitalier de Recherche Clinique*, PHRC AOM 09 180). There is no industry sponsorship or funding.

#### Coordination and conduct of the trial

Before recruitment of the first patient, all physicians and other healthcare workers in the 11 participating ICUs attended formal training sessions on the study protocol, data collection in the case-report form (CRF), and implementation of continuous EEG monitoring. All documents required for the study, including the study protocol and management guidelines, are available in each participating ICU. In each participating ICU, the physicians and a clinical research nurse and/or clinical research assistant are in charge of daily patient screening and inclusion, ensuring compliance with the study protocol, and collecting the study data in the CRF. Finally, all participating physicians are available for inclusion and continuous EEG monitoring around the clock and 7 days per week. The DRCD clinical research unit regularly reviews all clinical data collected in the CRF. Any serious adverse event must be reported within 48 h after its occurrence.

### Interim analyses

An interim analysis is scheduled after the enrolment of 135 patients. The O’Brien-Fleming method will be used.

### Blinding

Blinding of the physicians, nurses, and patients to the use of therapeutic hypothermia is not feasible. To minimise evaluation bias, outcomes will be evaluated during a structured phone interview by independent assessors blinded to the allocation group.

### Study outcomes

#### Primary outcome

The primary outcome is GOS score determined by a structured interview lower than 5 on day 90.

#### Secondary outcomes

Secondary outcomes are as follows:

- Percentages of patients with recurrent convulsive and/or non-convulsive seizures between 6 and 12 h after randomisation
- Total seizure duration in minutes
- Percentage of patients with refractory status epilepticus
- Mortality in the ICU, in the hospital, and by day 90
- Total ICU and hospital stay lengths in days
- Percentages of patients with functional impairments on day 90: frequency of seizures, recurrence of status epilepticus after discharge, number of antiepileptic drugs, MMSE score, and FIM score

### Modalities of outcome evaluation

The GOS score is determined on day 90 (±7 days) during a structured phone interview conducted by an independent assessor blinded to the allocation group (Table 2).

**Table 2:** Categories of the Glasgow Outcome Scale Adapted from Jennett B et al.

Category	Classification	Description
1	Death	Patient is certified dead
2	Vegetative state	Patient is unable to interact with the environment *Patients who show no evidence of meaningful responsiveness. This non*-*sentient state must be distinguished from other conditions of wakeful, reduced responsiveness*–*such as the locked*-*in syndrome, akinetic mutism, and total global aphasia. Vegetative patients breathe spontaneously, have periods of spontaneous eye*-*opening, may follow moving objects with their eyes, show reflex responses in their limbs (to postural or painful stimuli), and they may swallow food placed in their mouths*
3	Severe disability	Patient is unable to live independently but can follow commands *This indicates that a patient is conscious but needs the assistance of another person for some activities of daily living every day. This may range from continuous total dependency to the need for assistance with only one activity*
4	Moderate disability	Patient is capable of living independently but unable to return to work or school *Such a patient is able to look after himself at home, to get out, and about to the shops and to travel by public transport. However, some previous activities, either at work or in social life, are now no longer possible by reason of either physical or mental deficit*
5	Mild or no disability	Patient is able to return to work or school *This indicates the capacity to resume normal occupational and social activities, although there may be minor physical or mental deficits. However, for various reasons, the patient may not have resumed all his previous activities and in particular may not be working*

When possible, on day 90 (±7 days), patients return to the centre where they were included, for an evaluation by a neurologist including an interview and determination of the GOS score by structured interview, MMSE score, and FIM score. The patient brings an MRI of the brain and conventional EEG. The neurologist records any recurrent CSE episodes since randomisation; frequency of any recurrent seizures, as well as their type and associated symptoms; nature and dosage of antiepileptic drugs; and other treatments.

### Independent data safety monitoring board (DSMB)

The independent data safety monitoring board (DSMB) is composed of three physicians (Thomas De Broucker, Neurology Department, Saint Denis Hospital, Saint Denis, France; Alain Combes, Intensive Care Unit, Pitié Salpétrière University Hospital, France; and Nicolas Pichon, Intensive Care Unit, Limoges University Hospital, Limoges, France) and one biostatistician (Patricia Jabre, Urgences-Samu 93, Avicenne University Hospital, Bobigny, France) not otherwise involved in the trial. The DSMB assesses the safety data whenever complete 90-day follow-up data become available for 45 additional patients. For each of these safety analyses, the DSMB has access to blinded results on 28-day mortality, ICU discharge mortality, and 90-day GOS scores. The DSMB also evaluates all reported serious adverse events.

### End of the trial

Each patient is in the trial for 90 days. Premature study withdrawal is considered if requested by the patient or next of kin. Patients who are lost to follow-up or do not receive the randomly assigned treatment are not classified as prematurely withdrawn from the trial. Prematurely withdrawn patients are not replaced but undergo the procedures scheduled for the last visit, to the extent possible. Furthermore, the reason for premature withdrawal is recorded in both the CRF and the source document.

### Sample size

The working hypothesis for this randomised controlled trial is that, compared to standard care alone, standard care combined with therapeutic hypothermia increases the proportion of patients with a good 90-day outcome defined as a GOS score of 5. Assuming that 40 % of patients will have a GOS score of 5 on day 90 in the standard care group [4] and that this percentage will increase by 20 % (to 60 %) with therapeutic hypothermia, with a 5 % two-sided type I error rate, 90 % power, and a single interim analysis performed according to the O’Brien-Fleming method, 135 patients are needed in each group (270 patients in all).

### Statistical methods

The statistical analysis will follow the intention-to-treat approach, with each patient being analysed as a member of the group assigned by randomisation, regardless of subsequent events. In addition, any missing value will be replaced by the previous value according to the last value carried forward method. A sensitivity analysis of missing values will be carried out, using multiple imputation by chained equations (MICE). Comparisons of the two groups will routinely consider a possible centre effect.

A statistical analysis report will be written to describe all the findings according to CONSORT statement recommendations, taking into account the specific features of the trial. Any change in the analysis plan will be justified in the final report.

#### Primary outcome

The primary outcome will be assessed by using logistic regression to compare the percentage of patients with a 90-day GOS score lower than 5 in the two study groups. If needed, the logistic regression model will be adjusted to allow a more accurate assessment of the effect.

#### Secondary outcomes

The method described above for the primary outcome will also be used for the percentages of patients with EEG evidence of an ongoing epileptic seizure between 6 and 12 h or of refractory SE after randomisation; ICU, hospital, and 90-day mortality; and 90-day functional impairments.

To compare seizure duration (in minutes), we will build a semi-parametric regression model (Cox or Fine–Gray) appropriate for an analysis of competing risks (control of SE vs. death without control of SE). This approach will be used also for ICU and hospital stay lengths (alive at ICU or hospital discharge vs. death in the ICU or hospital, respectively).

## Discussion

SE is among the most common and challenging life-threatening events in patients with neurological failure. Despite national and international guidelines emphasising the importance of urgent anticonvulsant therapy according to a stepwise algorithm, SE remains associated with high mortality and morbidity rates. The contrast between the dual anticonvulsant and neuroprotective properties of therapeutic hypothermia demonstrated in experimental studies, on the one hand, and the paucity of clinical evidence, on the other, combined with the potential beneficial effects of therapeutic hypothermia on several causes of SE, prompted us to design the HYBERNATUS trial [35].

Several key points of the trial design received special attention and deserve discussion. First, our focus on patients with CSE requiring ICU management is warranted by reports of severe functional impairments after CSE. Indeed, the main objective of the study is neuroprotection, as opposed to only seizure control. We hypothesised that early implementation of our neuroprotective strategy in comatose patients after the control of CSE would translate into improved 90-day outcomes. We therefore decided to include not only patients with refractory CSE, but also all patients remaining comatose after control of motor seizures. Similarly, we planned to include patients regardless of the underlying aetiology even if this may lead to heterogeneity. As we were unwilling to require the use of mechanical ventilation for the trial, we included only patients who needed mechanical ventilation for any medical reason after CSE. Second, the choice of 32–34 °C as the therapeutic hypothermia target rests on both data from experimental animal models of SE and current practice regarding the use of therapeutic hypothermia in various conditions such as coma after cardiac arrest, stroke, or even traumatic brain injury [27, 36, 37]. Finally, the 24-h duration of therapeutic hypothermia after CSE control is based on the current standard of care for refractory SE, which includes anaesthetics for up to 24 h with a burst-suppression EEG pattern [5, 32]. Third, therapeutic hypothermia requires sedation and neuromuscular blockade. To ensure patient awakening as early as possible after the end of the neuroprotective procedure, we chose drugs with short half-lives, such as propofol and cisatracurium. Propofol has the additional advantage of exerting anticonvulsant effects. However, we felt it would be unethical to require the use of sedation in patients in the control arm. Thus, the continuation of sedation in controls is at the discretion of the bedside physician. Finally, safety considerations led us to limit the use of propofol to 48 h with an infusion rate no greater than 5 mg/kg/h, in both arms [38]. Fourth, we chose the 90-day GOS score as the primary outcome. The GOS is used chiefly to evaluate overall outcomes of neurological conditions [39–41]. The GOS in its structured form has been found valid, practical, and reliable [42]. It also has the advantage of having been used in several studies of SE, and it is well suited to an assessment of the physical and functional burden after CSE [43]. Moreover, in the preliminary work that allowed us to formulate the hypothesis for the sample size estimation, the 90-day functional outcome was evaluated using the GOS [4]. Importantly, the GOS has been found valid, practical, and reliable [42, 44].

To conclude, one of the main strengths of the HYBERNATUS trial is the uniformity of the study population and careful standardisation of CSE management in both groups. All patients are managed according to the recent literature, with continuous EEG monitoring and a comprehensive aetiological workup. The only difference between the two groups is the use of therapeutic hypothermia at 32–34 °C for 24 h, which is determined by randomisation. If the study hypothesis is confirmed, therapeutic hypothermia at 32–34 °C after control of CSE in patients requiring mechanical ventilation and ICU management will become part of standard practice in order to improve patient outcomes.

### Study protocol amendments and safety monitoring

Following first communication of a study indicating that moderate hypothermia may be harmful in patients with severe bacterial meningitis [45], we submitted a protocol amendment to the local independent ethics committee (Comité de Protection des Personnes CPP Ile de France IV, Saint Louis, on 15 January 2013) to not enrol patients with bacterial meningitis. This change was implemented on 24 January 2013.

Based on the results of planned the interim analysis (May 2013), the DSMB recommended the continuation of patients recruitment.

### Trial status

The trial has been validated and accepted for financial support by the French National Health Ministry (Programme Hospitalier de Recherche Clinique, PHRC AOM 09-P081249). Patient inclusion started in March 2011, and enrolments were completed on January 2015. Data management and planned analysis are ongoing, and release of the results is planned for the end of 2016.

## Abbreviations

CRF: case-report form
CSE: convulsive status epilepticus
EEG: electroencephalography
GOS: Glasgow Outcome Scale
ICU: intensive care unit
NCSE: non-convulsive status epilepticus
SE: status epilepticus
